# The effects of computerised cognitive training on post-CABG delirium and cognitive change: A prospective randomised controlled trial

**DOI:** 10.56392/001c.67976

**Published:** 2023-02-21

**Authors:** Danielle Greaves, Jack Astley, Peter J Psaltis, Amit Lampit, Daniel HJ Davis, Erica S Ghezzi, Ashleigh E Smith, Alice Bourke, Michael G Worthington, Michael J Valenzuela, Hannah AD Keage

**Keywords:** cognitive training, delirium, Coronary artery bypass grafting, cognition, post-operative

## Abstract

**Background:**

Cognitive impairments, including delirium, are common after coronary artery bypass grafting (CABG). Improving cognition pre- and post-operatively using computerised cognitive training (CCT) may be an effective approach to improve cognitive outcomes in CABG patients.

**Objectives:**

Investigate the effect of remotely supervised CCT on cognitive outcomes, including delirium, in older adults undergoing CABG surgery.

**Methods:**

Thirty-six participants, were analysed in a single-blinded randomised controlled trial (CCT Intervention: n = 18, Control: n = 18). CCT was completed by the intervention group pre-operatively (every other day, 45–60-minute sessions until surgery) and post-operatively, beginning 1-month post-CABG (3 x 45–60-minute sessions/week for 12-weeks), while the control group maintained usual care plus weekly phone calls. Cognitive assessments were conducted pre- and post-operatively at multiple follow-ups (discharge, 4-months and 6-months). Post-operative delirium incidence was assessed daily until discharge. Cognitive change data were calculated at each follow-up for each cognitive test (Addenbrooke’s Cognitive Examination III and CANTAB; z-scored).

**Results:**

Adherence to the CCT intervention (completion of three pre-operative or 66% of post-operative sessions) was achieved in 68% of pre-CABG and 59% of post-CABG participants. There were no statistically significant effects of CCT on any cognitive outcome, including delirium incidence.

**Conclusion:**

Adherence to the CCT program was comparatively higher than previous feasibility studies, possibly due to the level of supervision and support provided (blend of face-to-face and home-based training, with support phone calls). Implementing CCT interventions both pre- and post-operatively is feasible in those undergoing CABG. No statistically significant benefits from the CCT interventions were identified for delirium or cognitive function post-CABG, likely due to the sample size available (study recruitment greatly impacted by COVID-19). It also may be the case that multimodal intervention would be more effective.

## Introduction

Coronary artery bypass grafting (CABG) is the most performed cardiovascular surgery worldwide.^1^ Although CABG is known to improve cardiac function and has low rates of surgery-related mortality,^2^ it is commonly associated with cognitive decline and delirium.^3^ The impact of these cognitive outcomes on a patient’s prognosis is well documented, including increased risk of dementia, mortality, early retirement, readmissions and dependency, decreased quality of life and physical function.^4–11^

Potentially modifiable risk factors for post-CABG delirium and cognitive decline exist including pre-operative depression, diabetes, hypertension and cognitive impairment.^12^ Most CABG patients have short lead-up times (less than a month) to surgery, rendering some modifiable risk factors difficult to address. Improving cognition through computerised cognitive training (CCT) is an attractive target for pre-operative intervention as it is a scalable, adaptable, relatively inexpensive and safe approach, known to be effective following relatively short interventions (e.g. 2-3 weeks, completing 6-9 hours of training).^13–15^

Post-operative cognitive training in cardiac and noncardiac patients has elicited improvements in trained domains of memory (including working memory)^16–18^ and attention,^18^ as well as reductions in depression and anxiety.^17^ In pre-operative settings (non-cardiac patients), cognitive training has significantly reduced cognitive decline (1-week post-operatively)^19^ and delirium incidence^20^ when compared with controls. Other studies reported no significant benefits in cardiac and non-cardiac patients,^21,22^ albeit with small sample sizes and sub-optimal intervention designs (e.g. unsupervised, short sessions).^23^ Beneficial effects may be plausible with a more optimally designed CCT program including supervision and less regular and longer sessions,^23^ which have been implemented in the current study.

The current paper investigates the effect of remotely supervised CCT on cognitive outcomes following CABG surgery in older adults. Specifically, our primary aims are to determine the effect of 1) pre-CABG CCT on cognitive function at discharge and the incidence and severity of post-operative delirium and 2) pre- and post-CABG CCT on cognitive function at 4, and 6- months.

## Methods

A detailed description of the study protocol, including the timing of all study measurements, has been published.^24^ In brief, the study was a single-blinded, randomised controlled trial with longitudinal follow-up comparing pre- and post-operative CCT (intervention group) with usual care (control group). Persons scheduled to undergo CABG or CABG plus a concomitant surgery, with at least one-week lead-up time before surgery, who were aged ≥65 years, spoke English proficiently and lived within a 1-hour drive from the Adelaide (South Australia) central business district were approached. As detailed in the published protocol paper,^24^ the target sample size was n=120. However, recruitment was severely impacted due to COVID-19 restrictions beginning in 2020, with the cancellation of elective procedures within the South Australian hospital system, resulting in scheduled surgeries of metropolitan patients being completed in private hospitals (not covered by ethical approval).

**Figure 1:**
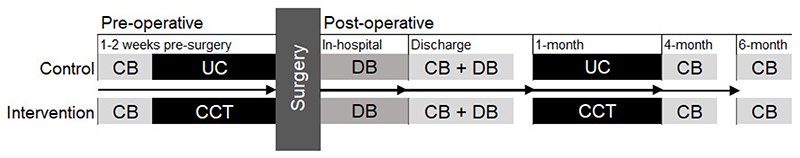
Study timeline highlighting the time of assessments and interventions, specific to control and intervention. Assessment sessions contain cognitive battery and other additional assessments. CB, cognitive battery; CCT, computerised cognitive training; DB, daily delirium battery in hospital; UC, usual care.

Once randomised (using a randomisation list^24^) and consented (note participants received a group-specific information sheet and consent form), cognitive function and depression were measured before surgery (baseline), at discharge, as well as post-operatively at 4- (end of intervention) and 6-months. The presence of delirium was measured post-operatively; in-hospital (daily) and at discharge (see Figure 1). All cognitive and delirium assessments were performed by trained researchers who were blinded to the participant’s group allocation.

### Delirium Assessment Battery

The intensive care unit (ICU) Confusion Assessment Method (CAM-ICU)^25^ and the Memorial Delirium Assessment Scale (MDAS) interview^26^ (which informed the scoring of the CAM^27^) were conducted daily when participants were in the ICU and ward respectively. In addition, chart reviews were utilised to ascertain delirium presence^28^ over the weekend. In the case of a positive delirium diagnosis (CAM positive or delirium documented in the medical record) the participant was coded as having delirium in those 24-hours. Delirium severity was quantified using the CAM-ICU 7^29^ (when in the ICU) and the MDAS (when in the ward).

### Cognitive Assessment Battery

Global cognitive status was measured utilising the Addenbrookes Cognitive Examination (ACE-III).^30^ Cognition was assessed with a computerised battery (Cambridge Neuropsychological Test Automated Battery (CANTAB) Connect).^31^ Specific CANTAB tests included Reaction Time (RTI), Paired Associates Learning (PAL), Spatial Working Memory (SWM) and One Touch Stockings of Cambridge (OTS). For each CANTAB test, at least two outcome measures (e.g., median reaction time for correct responses) were extracted for analyses; see Supplementary Table 1 for brief descriptions of outcome measures of interest. Depression was measured using the Geriatric Depression Scale (Short Form).^32^

### Computerised Cognitive Training Intervention

CCT was delivered through the HappyNeuron Pro platform (on a laptop), with selected exercises targeting cognitive domains most affected in heart failure: psychomotor speed, attention, memory and executive function.^33–36^ For the intervention group, CCT sessions lasting 45-60 minutes were completed every other day pre-operatively, and three times a week post-operatively (beginning 1-month post-operatively, continuing for 12 weeks). During intervention periods, participants were offered at least one completely supervised session weekly, face-to-face or remotely (via TeamViewer), with a trained researcher (trained by AL). In addition, participants were contacted weekly via phone (offering support, encouragement, or technical help along with a measurement of pain). Control participants did not complete any CCT but were contacted weekly via phone (for a pain measure, data not included here). Adherence, identified through app metrics, was considered acceptable if three pre-operative sessions were completed, or 66% of the scheduled post-operative sessions were completed. Those who did not adhere pre-operatively could still be considered adherers for the 4- and 6-month follow-ups. Only data from adherers were used in analyses.

### Statistical Analyses

All analyses were performed using R (version 4.2.0)^37^ using the Rstatix package.^38^ As described in the published protocol paper, a per-protocol approach was utilised,^24^ due to the importance of investigating the efficacy of the intervention as intended (only participants who reached adherence criteria were included for analyses). Due to the limited sample size, effect sizes were produced for each analysis to interpret potential findings, rather than focusing on statistical significance.

### Primary Analysis

A logistic regression was performed to assess the effect of CCT (intervention=1, control=0) on delirium (presence=1, absence=0), with age and sex as covariates. An additional sensitivity analysis was run including baseline cognition (ACE-III) and depression (GDS) as covariates.

### Primary Cognitive Change Analysis

ACE-III raw scores at the three follow-ups were subtracted from baseline scores to index change in global cognition over time. Independent samples t-tests were conducted assessing the effect of CCT on the change in test performance at each follow-up.

CANTAB test scores were used to assess domain-specific cognition. Z-scores were calculated for each CANTAB test outcome measure (see Supplementary Table 1) at baseline and each of the three follow-up timepoints. All z-scores were calculated based on the respective measure’s baseline distribution to ensure change was indexed appropriately.^39^

$$z=\frac{x^{raw\;score\;at\;follow\;-up\;}-\mu^{baseline\;}}{\sigma^{baseline\;}}$$

Where necessary, scores were reversed to ensure consistency. Higher numbers (z-scores) reflected better performance. Average composite z-scores were calculated for each test across the selected outcome measures. Each composite follow-up z-score was subtracted from the composite baseline z-scores to index change in test performance over time. Independent samples t-tests were conducted assessing the effect of CCT on the change in test performance from baseline to the three timepoints. Cohen’s d values were calculated as measures of effect size. Multiple comparisons were not adjusted for due to the small/restricted sample size

### Exploratory Delirium Analysis

A linear regression was performed to assess the effect of CCT on delirium duration (number of days delirious), with age and sex as covariates. This included participants who did not experience delirium (0 days).

Two separate linear regressions were performed assessing the effect of CCT on delirium severity; 1) delirium severity in the ICU (CAM ICU-7), 2) delirium severity in the ward (MDAS). Both regressions included age and sex as covariates. Unstandardised beta values (B) were used to indicate effect size and direction. Distributions of the residuals of each regression indicated that model assumptions were not violated.

## Results

See CONSORT diagram (Supplementary Figure 1) for participant flow through the study. 45 participants consented and data from 36 participants were analysed (18 per study group). Of those randomised to the CCT group (n= 24), 68% adhered pre-operatively and 59% adhered post-operatively (see Table 1 for demographic, cognitive and delirium data breakdown by study group). 42% of participants experienced delirium after surgery.

### Incident Delirium Outcomes

Pre-operative CCT did not significantly associate with delirium following CABG surgery (OR=1.25, 95%CI=[0.30, 5.24], *p*=0.76), covarying for age and sex. Age and sex also did not significantly associate with incident delirium. Model 2, which included baseline global cognition and depression, did not yield any significant effects (Table 2).

### Cognition Outcomes

**Figure 2:**
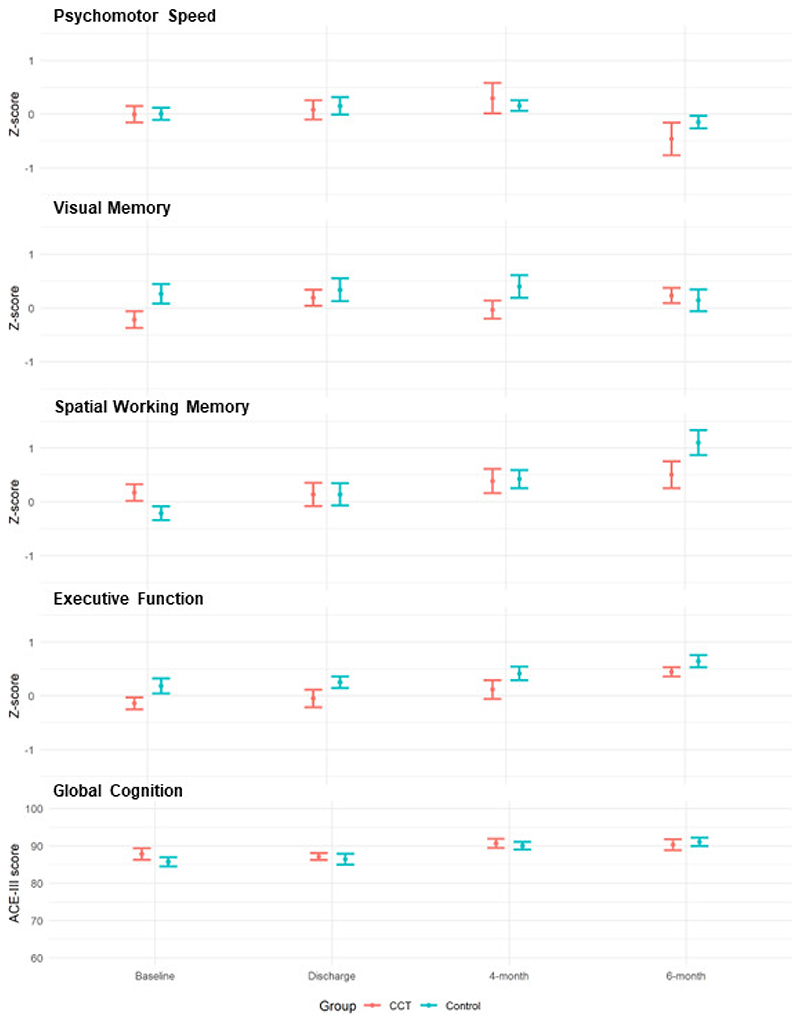
Global and domain-specific cognition performance over time, stratified by CCT and Control groups. Means and standard errors indicated by error bars. Z-scores were calculated using baseline distribution of each test. Higher z-scores indicate better change in performance from baseline to follow-up

There were no significant effects of CCT on the change in global cognition performance (ACE-III) at discharge, 4- and 6-month follow-ups. There were also no significant effects of CCT on the change in performance on any cognitive domains (CANTAB) at discharge, 4- and 6-month follow-ups (Figure 2 and Supplementary Table 2 and 3).

### Exploratory Delirium Severity And Duration Analysis

Pre-operative CCT did not significantly associate with the duration of post-operative delirium (B=-0.79, p=0.32, 95%CI=[-2.31, 0.74]), while covarying for age and sex. Pre-operative CCT did not predict the severity of delirium in the ICU (B=0.25, p=0.58, 95%CI=[-0.62, 1.11]) or on the ward (B=-0.30, p=0.79, 95%CI=[-2.50, 1.91]), covarying for age and sex. Age significantly predicted delirium severity in the ICU (B=0.08, p=0.04, 95%CI=[0.01, 0.16]), with older participants experiencing more severe delirium. Sex did not associate with delirium severity in the ICU. Age and sex also did not significantly predict delirium duration or severity on the ward, (Table 3).

## Discussion

We saw no meaningful effects of CCT on incident delirium and cognitive function in CABG patients. Notably, our study is underpowered, due to substantial difficulties in recruiting CABG patients during the COVID-19 pandemic. Exploratory analyses revealed age as a predictor of increased delirium severity in the ICU, yet, this is a known effect.^29^

The findings indicate that pre- and post-operative CCT interventions, which include participant-tailored support (e.g., additional face-to-sessions and weekly phone calls), are feasible for CABG patients. In the CCT group, adherence was seen in 68% of participants pre-operatively. This rate is comparatively higher than those reported in recent feasibility trials (ranging from 17 to 39%) investigating the effect of pre-operative CCT on post-operative delirium and cognitive decline.^21,22^ These trials prescribed short (15-20 minutes), frequent (up to twice daily), unsupervised and home-based CCT sessions. This may have driven their lower adherence rates, with participants reporting feeling over-whelmed.^21,22^ In contrast, with our approach (45-minute sessions, maximum frequency of every other day, with support) participants in a qualitative sub-study reported “it’s manageable but with support”.^40^ This highlights that support from our research staff (starting sometimes with how to use a computer) was essential in overcoming the perceived barriers of completing CCT, and is likely a driving factor in the adherence rate disparity to the previous feasibility trials.

Although adherence was considerably more favourable compared to previous trials there were no statistically significant differences between study groups concerning the incidence, severity, and duration of delirium post-operatively. These findings are similar to previous feasibility tri-als.^21,22^ However, a recent large randomised controlled trial (the Neurobics trial n=251) found that pre-operative CCT (utilising the Lumosity platform) significantly lowered post-operative delirium risk (RR=0.58, *p*=.047) in cognitively healthy, non-cardiac surgical patients, who were at least minimally compliant to the CCT intervention.^20^ Although minimum lead-in times to surgery were similar between Neurobics and the current study (one week), CCT was prescribed more frequently in the former (daily pre-operatively; suggested minimum 1-hour daily, target of 10-hours pre-operatively). Therefore, hours of completed CCT were likely higher in the Neurobics trial.^20^ Although, it is known that CCT delivered less frequently (≤ 3 sessions a week) is more effective than more frequent sessions across weeks to months of an intervention.^23^

There were no statistically significant differences between CCT and control groups, relative to change in performance in cognitive domains (individual CANTAB tests) or global cognition (ACE-III), from baseline to discharge, 4- or 6-month follow-ups. Within the literature, meaningful cognitive change for an individual (commonly investigated as decline) is characterised by a 1 standard deviation change at a post-operative follow-up compared to pre-operative performance.^41^ This change (with a non-significant large effect, *d=* 0.83) at a group level only occurred in the control group at 6-month follow-up (as compared to baseline) in a task targeting executive function (SWM). However, this result is likely driven by low baseline scores in the control group in this task. The CCT intervention focused on executive function (e.g. goal formulation, planning, strategy). However, recent meta-analyses revealed CCT brings about only negligible-to-small improvements in this domain , which do not reach statistical significance, for both healthy older adults^23^ and those with mild cognitive im-pairments.^42^

A meta-analysis, containing data of over 2,000 CABG patients, reported a trend towards improvement in overall cognitive function across early (3-6 months) and late (6-12 months) follow-ups compared to pre-operative performance.^43^ This same pattern can be seen in our data (see Figure 2), where group-level performance almost always improves across all cognitive measures (excluding reaction time), especially at 4- and 6-month follow-ups. This improvement could be driven by increased blood perfusion in the brain resultant of improved functional cardiac output following surgery.^44^ Further, our follow-up sample may have been positively biased (more cognitively healthy), due to an increased withdrawal rate in those who experienced delirium. The current study investigated at a group level, comparing control with CCT participants. With more statistical power (participant numbers), it would have been ideal to investigate how CCT affected known post-operative cognitive trajectories: decline, no change, and improvement. It may be that the pattern of general cognitive improvement is masking different patterns and combinations of cognitive trajectories relative to group.

## Limitations

Due to the small sample size available for analyses, the study is not sufficiently powered. Notably, we did not see any patterns of effect sizes (rather than statistical significance) across outcomes that supported our original hypotheses. The current study was limited by a two-group design. Consequently, the independent effects of pre-CABG and post-CABG CCT could not be investigated. In addition to this, no sham control was utilised within this study. However, this is not a considerable limitation due to; 1) the use of separate consent forms for study groups (i.e. neither knew of the alternate comparison group), and 2) a meta-analysis investigating the effect modifiers of CCT in cognitively healthy older adults found no significant effect of control group (passive versus active).^23^ These analyses were conducted utilising data from CABG patients recruited from a single-centre hospital based in metropolitan South Australia, limiting the ability to generalise results.

## Future Directions And Conclusion

This study provides support for the feasibility of implementing CCT interventions pre- and post-operatively in cardiac surgical settings. Given the reported null effects, it is possible CCT may not be an effective method for reducing post-CABG delirium and cognitive decline; or null effects could be due to our small sample. As there are reported small protective effects of CCT on delirium incidence,^20^ further research (within larger samples) is warranted. This study focused on a single-mode intervention (CCT). Multicomponent interventions, where multiple risk factors are targeted concurrently within the one intervention, are another viable method of pre-operative intervention shown to be beneficial in improving (or maintaining) cognition in those at risk of dementia and reducing incident delirium during hospitalisation.^45–47^

## Supplementary Material

Supplementary Material

## Figures and Tables

**Table 1:** Baseline demographic, cognitive and delirium characteristics, relative to each study group (CCT and control).

	CCT			Control			Total		
	N	Mean (%)	SD	N	Mean (%)	SD	N	Mean (%)	SD
Age	18	73.8	6.4	18	72.6	5.7	36	73.2	6
Sex	18			18			36		
F	4	(22%)		2	(11%)		6	(17%)	
M	14	(78%)		16	(89%)		30	(83%)	
ACE-III	18	88	8.4	18	85.7	7	36	86.9	7.7
GDS	18	2.6	1.8	18	2.8	2.9	36	2.7	2.4
Delirium*	15			18			33		
No	8	(53%)		11	(61%)		19	(58%)	
Yes	7	(47%)		7	(39%)		14	(42%)	
Days Delirious*	15	0.9	1.3	18	1.5	2.7	33	1.2	2.2
CAM-ICU*	15	1.8	1.5	18	1.3	1.2	33	1.5	1.3
MDAS*	15	5.1	3.1	18	5.5	3.1	33	5.3	3

* indicates sample number included in delirium analysis only (pre-operative CCT adherers). Please note, different participants may be included in each analysis due to adherence being considered separately for the CCT pre- and post-operatively. ACE-III = Addenbrookes Cognitive Examination III, CCT = computerised cognitive training, GDS = Geriatric Depression Scale, CAM-ICU = The intensive care unit Confusion Assessment Method, MDAS = Memorial Delirium Assessment Scale, F = female, M = male, N= number of participants in analysis.

**Table 2:** Results of logistic regression models assessing the effect of CCT on incident delirium.

	OR	95% CI	p
Model 1 (N=33)			
CCT (CCT = 1, Control = 0)	1.25	0.30, 5.24	0.76
Age	1.03	0.91, 1.18	0.62
Sex (Male = 1, Female = 0)	0.87	0.13, 6.13	0.90
Model 2 (N=33)			
CCT (CCT = 1, Control = 0)	1.47	0.32, 6.65	0.62
Age	1.00	0.83, 1.16	0.98
Sex (Male = 1, Female = 0)	0.63	0.07, 5.48	0.68
Global cognition	0.95	0.84, 1.07	0.37
Depression	1.05	0.76, 1.43	0.79

Note: *Model 1 includes age and sex as covariates, model 2 includes age, sex, global cognition (Addenbrookes Cognitive Examination - III) and depression (Geriatric Depression Scale) as covariates.* CCT = computerised cognitive training.

**Table 3:** Results of linear regressions assessing the effect of CCT on delirium duration (days delirious) and delirium severity in the ICU and ward.

Delirium Duration (n=33)	*B*	SE	95% CI	*t*	*p*
CCT (CCT = 1, Control = 0)	-0.79	0.78	-2.31, 0.74	-1.01	0.32
Age	0.13	0.07	-0.01, 0.27	1.86	0.07
Sex (Female = 0, Male = 1)	0.65	1.07	-1.44, 2.74	0.61	0.55
Delirium Severity – ICU (n=33)					
CCT (CCT = 1, Control = 0)	0.25	0.44	-0.62, 1.11	0.56	0.58
Age	0.08	0.04	0.01, 0.16	2.13	0.04
Sex (Female = 0, Male = 1)	-0.44	0.60	-1.62, 0.74	-0.73	0.47
Delirium Severity – Ward (n=33)					
CCT (CCT = 1, Control = 0)	-0.30	1.12	-2.50, 1.91	-0.26	0.79
Age	0.07	0.10	-0.13, 0.27	0.68	0.50
Sex (Female = 0, Male = 1)	1.55	1.54	-1.46, 4.56	1.01	0.32

Note: *Age and sex were included as covariates in all three models.* CCT = computerised cognitive training.
