# Supplementary material for: The effects of computerised cognitive training on post-CABG delirium and cognitive change: A prospective randomised controlled trial
Source: Delirium (Bielef). Author manuscript; Available in PMC 2023 Mar 17. (PMC7614332; doi:10.56392/001c.67976)
Supplement: Supplementary Material [file EMS172111-supplement-Supplementary_Material.docx]

Supplementary Table 1. CANTAB test and outcome measure descriptions (including the direction of effect for each outcome measure) utilised within cognitive decline analyses.

| **Name (test/outcome measure)** | **Description (test/outcome measure)** |
| --- | --- |
| Reaction Time (RTI) test | The participant must select and hold a button at the bottom of the screen. Circles are presented above (one for the simple mode, and five for the five-choice mode). In each case, a yellow dot will appear in one of the circles, and the participant must react as soon as possible, releasing the button at the bottom of the screen, and selecting the circle in which the dot appeared. |
| RTI outcome measures |  |
| Simple median reaction time | Time in milliseconds from circle presentation to button release for correct trials (only simple mode trials). |
| Simple median movement time | Time in milliseconds from button release to selecting the target circle for correct trials (only simple mode trials). |
| Five-choice median reaction time | Time in milliseconds from circle presentation to button release for correct trials (only five-choice mode trials). |
| Five-choice median movement time | Time in milliseconds from button release to selecting the target circle for correct trials (only five-choice mode trials). |
| Paired Associates Learning (PAL) test | Boxes are displayed on the screen and are “opened” in a randomised order. One or more of them will contain a pattern (assessed on 4 to 12 patterns). The patterns are then displayed in the middle of the screen, one at a time and the participant must select the box in which the pattern was originally located. If the participant makes an error, the boxes are opened in sequence again to remind the participant of the locations of the patterns. |
| PAL outcome measures |  |
| Total errors (adjusted) | The number of times an incorrect box was selected, plus an adjustment for the estimated number of errors that would have been made on any problems, attempts, and recalls in levels they did not reach. |
| First attempt memory score | The number of times the correct box was selected on their first attempt when recalling the pattern locations. |
| Spatial Working Memory (SWM) test | The test begins with several coloured squares (boxes) shown on the screen (ranges from 4-12). The aim of this test is that by selecting the boxes and using a process of elimination, the participant should find one yellow ‘token’ in each box (a token will never be in the same box twice) and use them to fill up an empty column on the right-hand side of the screen. |
| SWM outcome measures |  |
| Between errors | The number of times a box is selected in which a token has previously been found. Calculated across all assessed four, six and eight token trials. |
| Strategy | The number of times a new search pattern is started. If they always begin a search from the same starting point, this infers a planned strategy for finding the tokens. Therefore, low scores indicate high strategy use (i.e. 1 = they always begin the search from the same box). Calculated across assessed trials with 6-8 tokens. |
| One-Touch Stockings of Cambridge (OTS) test | The participant is shown two displays containing three coloured balls. There is a row of numbered boxes along the bottom of the screen. The test administrator (I-pad) demonstrates to the participant how to move the balls in the lower display to copy the pattern in the upper display. Following three practice trials, the participant is shown further problems and must work out in their head how many moves the solutions require, and then select the appropriate box at the bottom of the screen to indicate their response. |
| OTS outcome measures |  |
| Problems solved on first choice | The total number of assessed trials where the correct answer was selected on the first attempt. Calculated across all assessed trials. |
| Median latency to first choice | The median latency, from the appearance of the stocking balls until the number of moves selected. Calculated across all assessed trials where the first response was correct. |

*Note: Global Cognition = Addenbrookes Cognitive Examination performance, Executive function = One Touch Stockings of Cambridge performance, Visual memory = Paired Associates Learning performance, Psychomotor Speed & Attention = Reaction Time performance.*

*Supplementary Table 2. Results of independent samples t-tests assessing the effect of CCT on the change in global and domain-specific cognition at the discharge, 4-month (4m) and 6-month (6m) follow ups.*

| ACE-III | n (CCT) | n (Control) | t | DF | *p* | *d* |
| --- | --- | --- | --- | --- | --- | --- |
| Global Cognition Discharge | 13 | 16 | 0.25 | 25.70 | 0.81 | 0.09 |
| Global Cognition 4m | 12 | 16 | 0.26 | 21.60 | 0.80 | 0.10 |
| Global Cognition 6m | 12 | 13 | -0.26 | 18.90 | 0.80 | -0.11 |
| CANTAB |  |  |  |  |  |  |
| Executive Function Discharge | 11 | 14 | -0.08 | 18.79 | 0.94 | -0.03 |
| Executive Function 4m | 12 | 14 | -0.43 | 17.82 | 0.67 | -0.17 |
| Executive Function 6m | 11 | 11 | 0.88 | 13.95 | 0.39 | 0.39 |
| Visual Memory Discharge | 12 | 14 | 1.34 | 22.76 | 0.20 | 0.53 |
| Visual Memory 4m | 12 | 14 | 0.31 | 23.43 | 0.76 | 0.12 |
| Visual Memory 6m | 12 | 13 | 0.98 | 21.02 | 0.34 | 0.40 |
| Psychomotor Speed & Attention Discharge | 13 | 16 | 0.43 | 22.95 | 0.67 | 0.16 |
| Psychomotor Speed & Attention 4m | 12 | 14 | 0.39 | 13.32 | 0.70 | 0.16 |
| Psychomotor Speed & Attention 6m | 12 | 13 | -0.88 | 13.71 | 0.40 | -0.37 |
| Spatial Working Memory Discharge | 12 | 15 | -1.76 | 21.90 | 0.09 | -0.66 |
| Spatial Working Memory 4m | 12 | 14 | -1.29 | 19.75 | 0.21 | -0.51 |
| Spatial Working Memory 6m | 12 | 13 | -2.03 | 21.96 | 0.06 | -0.83 |

*Note: Global Cognition = Addenbrookes Cognitive Examination performance, Executive function = One Touch Stockings of Cambridge performance, Visual memory = Paired Associates Learning performance, Psychomotor Speed & Attention = Reaction Time performance.*

|  | Mean Baseline | SD Baseline | Mean Discharge | SD Discharge | Mean 4-month | SD 4-month | Mean 6-month | SD 6-month |
| --- | --- | --- | --- | --- | --- | --- | --- | --- |
| Global Cognition | 86.8 | 7.66 | 86.58 | 7.53 | 89.65 | 6.48 | 90.78 | 7.01 |
| Psychomotor Speed & Attention | 0 | 0.77 | 0.11 | 0.97 | 0.23 | 1.25 | -0.31 | 1.32 |
| Visual Memory | 0 | 0.98 | 0.26 | 1.03 | 0.16 | 1.09 | 0.19 | 0.98 |
| Spatial Working Memory | 0 | 0.84 | 0.14 | 1.2 | 0.4 | 1.14 | 0.79 | 1.39 |
| Executive Function | 0 | 0.72 | 0.1 | 0.8 | 0.26 | 0.88 | 0.54 | 0.56 |

*Supplementary Table 3. Means and standard deviations of raw global cognition scores and composite z-scores for domain-specific cognition at baseline and the discharge, 4-month (4m) and 6-month (6m) follow ups.*

*Note: Global Cognition = Addenbrookes Cognitive Examination performance, Executive function = One Touch Stockings of Cambridge performance, Visual memory = Paired Associates Learning performance, Psychomotor Speed & Attention = Reaction Time performance.*

Supplementary Figure 1: CONSORT diagram presenting the reasoning for participant data exclusion at each stage of the study.
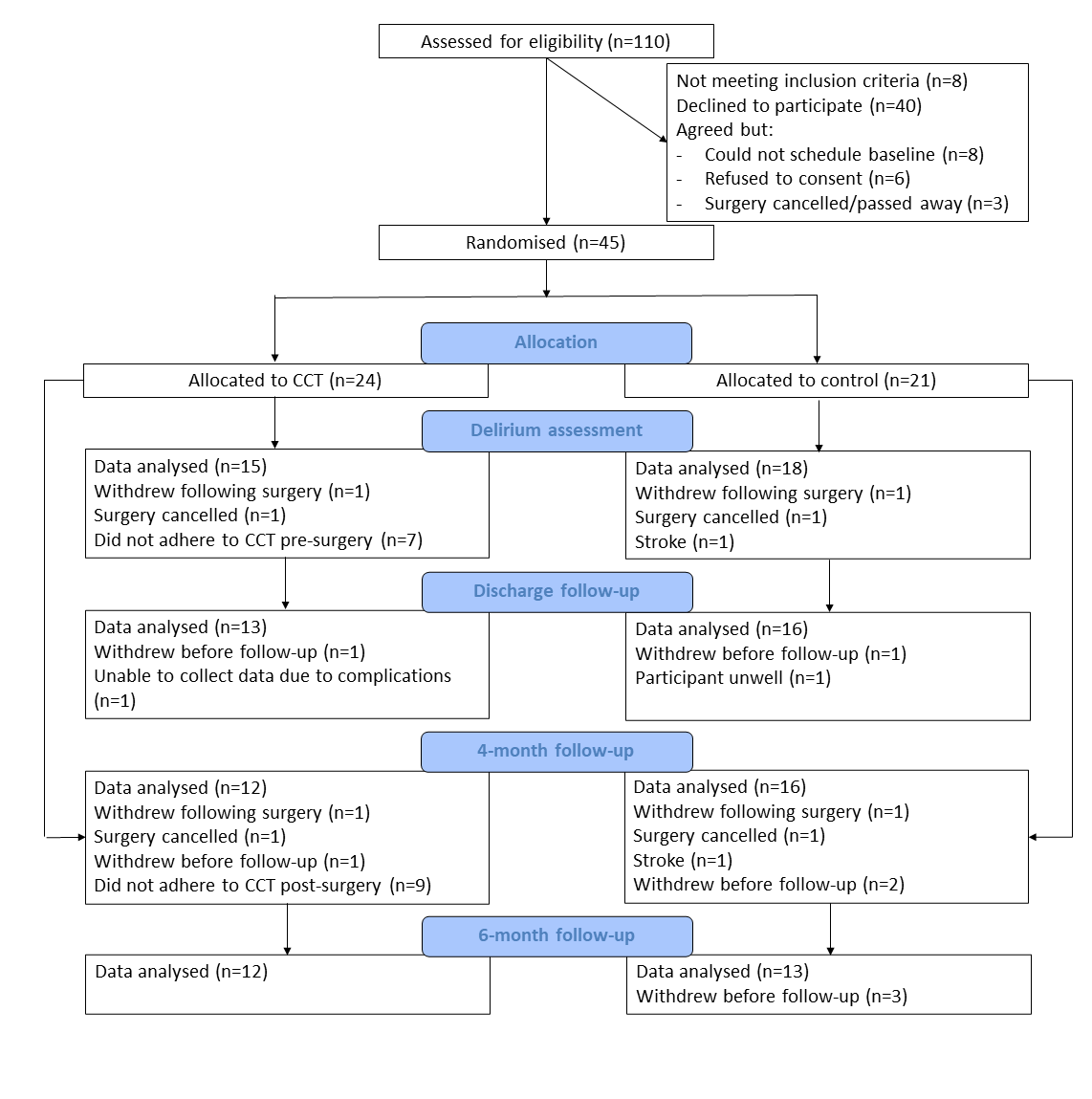


*Participants who adhered pre-operatively were included for data analysis at the delirium assessment and discharge follow-up. Participants who adhered post-operatively were included for data analysis at the 4- and 6-month follow-ups.* *Please note, different participants may be included in each analysis due to adherence being considered separately for the CCT pre- and post-operatively.* *36 participants were analysed in total, adhering either pre- or post-operatively. CCT= Computerised Cognitive Training*
